# A review method for UML requirements analysis model employing system-side prototyping

**DOI:** 10.1186/2193-1801-2-134

**Published:** 2013-03-26

**Authors:** Shinpei Ogata, Saeko Matsuura

**Affiliations:** Department of Computer Science and Engineering, Facluty of Engineering, Shinshu University, Nagano, Nagano, 380-8553 4-17-1 Wakasato, Japan; Division of Electrical Engineering and Computer Science, Graduate School of Engineering, Shibaura Institute of Technology, 307 Fukasaku, Minuma-ku, Saitama, Saitama, 337-8570 Japan

**Keywords:** Business logic, Check-list, Prototyping, Object-oriented analysis, Review, Unified modeling language

## Abstract

User interface prototyping is an effective method for users to validate the requirements defined by analysts at an early stage of a software development. However, a user interface prototype system offers weak support for the analysts to verify the consistency of the specifications about internal aspects of a system such as business logic. As the result, the inconsistency causes a lot of rework costs because the inconsistency often makes the developers impossible to actualize the system based on the specifications. For verifying such consistency, functional prototyping is an effective method for the analysts, but it needs a lot of costs and more detailed specifications. In this paper, we propose a review method so that analysts can verify the consistency among several different kinds of diagrams in UML efficiently by employing system-side prototyping without the detailed model. The system-side prototype system does not have any functions to achieve business logic, but visualizes the results of the integration among the diagrams in UML as Web pages. The usefulness of our proposal was evaluated by applying our proposal into a development of Library Management System (LMS) for a laboratory. This development was conducted by a group. As the result, our proposal was useful for discovering the serious inconsistency caused by the misunderstanding among the members of the group.

## Introduction

Defects of a requirements specification for a business software bring on disastrous failure for the software development project because the defects can consume 70 percentage to 85 percentage of all project rework costs (Wiegers 2003). Therefore, it is important to validate and verify the requirements specification sufficiently at the requirements analysis phase. User interface prototyping (ACM 1982) is an effective method to facilitate users’ validation to the requirements specification. Analysts often make the users confirm the requirements specification by using a user interface prototype system at an early stage of a development for an interactive software such as a business application because the users can understand the requirements intuitively by operating the user interface. We have proposed a method for generating a user interface prototype system from Unified Modeling Language (UML) requirements analysis (RA) model (Ogata and Matsuura 2008;2010) so that the analyst can accept the advantage of the user interface prototype system easily. This UML RA model is a kind of a use-case-based model and consists of activity diagrams, a class diagram and object diagrams. However, the user interface prototype system can not visualize internal aspects of a system such as business logic. Therefore, the analysts can not verify the requirements specification about the internal aspects efficiently. On the other hand, a functional prototype system enhances the efficiency of the analysts’ verification because the analysts can confirm the response of the system actually to inputs via implemented functions and functions. However, the cost for the functional prototyping is expensive because more implementation and detailing are needed. Here, more implementation implies that the functions and methods for the functional prototype system actually are implemented. More detailing also implies that the specification corresponding with the functional prototype system is detailed. If an invalid functional prototype system is created based on the misunderstanding among the analysts to the specification, few of the large costs which were spent for creating such functional prototype system will contribute to the product directly. Moreover, it is difficult to prevent such inconsistency in a development by a group because the members of the groups may not share the comprehension to the requirements specification sufficiently. Especially, the part of the specification which a certain member of the group manages directly is hard to be understood by the rest of the members who do not manage the part directly. To improve such problems, we propose a review method based on the UML RA model for clearing the misunderstanding among analysts about the definition of business logic so that the analysts can discover such inconsistency among each diagram sufficiently and intuitively before detailing the specification and creating a functional prototype system or product. We also propose a check-list and system-side prototyping used in this review method. The check-list is for discovering two kinds of inconsistencies in the activity diagrams mainly. One is mismatches of pre/post-conditions between actions. The other is inadequate relations between an action and objects. The check-list is used by each analyst so that he can understand his part of the UML RA model precisely. The system-side prototype system does not have any functions to achieve business logic, but visualizes the result of integration among three kinds of the diagrams in the UML RA model. The system-side prototype system is used when the analysts of a group share and verify each part of the specification managed by each of the analysts. The usefulness of our proposal was evaluated by applying our proposal to a development of Library Management System (LMS) for a laboratory. As the result, the analysts could discover the serious inconsistent interpretations of the UML RA model which were caused by the misunderstanding among the members of the group. In “Background” section, we introduce the necessary of our proposal and compare our proposal with related work. In “Method” section, we explain our proposal based on the UML RA model described as “The UML RA model” section. Then, we explain the result of an evaluation of the effectiveness of our proposal in “Results and discussion” section. Finally, we describe the conclusion of this paper in “Conclusion” section.

## Background

The following points are important in order to develop the enterprise system so as to satisfy the requirements of users.

1. A way to validate a requirements specification should be shaped so that the users can validate the requirements specification sufficiently and intuitively because the users are not experts of a software development generally. User interface prototyping is one of the effective methods for validating the requirements specification by the users sufficiently.
2. IEEE 830 (IEEE 1998), which is an international standard for requirements specification, recommends that the requirements specification should contain not only the external aspects such as user interfaces but also internal aspects of a system such as business logic. We call the internal aspects of a system as the internal aspects simply in this paper. Therefore, analysts must define the business logic so that software designers and test planners can sufficiently and precisely understand the requirement specification about the internal aspects of the system under development.

To support the validation and verification for the requirements specification, there are a lot of researches (Choi and Watanabe 2005; Diaz et al. 1996; Elkoutbi et al. 2006; Thelin et al. 2003). However, even if the methods above-mentioned researches propose are applied into requirements analysis, it is difficult to verify the internal aspects sufficiently. We explain its reasons at next two subsections.

### The misunderstanding of specifications in group work

A lot of developments are conducted by groups or organizations. It is impossible for each member to understand all of the software specification in such group work. Therefore, the various consensus-building processes such as assessment, review and testing is conducted constantly between users and developers or between analysts and designers, etc. The user interface prototyping is an effective method for users to validate the requirements specification about users’ operation efficiently and intuitively, but the users can not confirm the validity of the internal aspects mostly. Therefore, the users often agree the requirements specification through user interface prototype system even if the system can not be actualized based on the specification. On the other hand, the more a development becomes large-scale, the more analysts are needed. In a large-scale development, the misunderstandings among analysts to the specification will cause serious reworking because invalid product is created based on the inconsistent specification as the result of the misunderstandings. Therefore, it is important for each analyst to have common understanding of the specification among all of the analysts sufficiently so as to prevent defining inconsistent specifications. Support of the verification of the specification without detailing is also necessary for analysts so that the analysts can clear the misunderstanding to the specification constantly before causing the serious reworking. The methods for generating a user interface prototype system from a requirements specification (Diaz et al. 1996; Elkoutbi et al. 2006) are useful for analysts to make users validate the external aspects of the system. (Elkoutbi et al. 2006) proposed how to write use cases by using UML collaboration diagrams, a UML class diagram and UML state machine diagrams. (Diaz et al. 1996) also proposed how to write use cases by using a UML use case diagram, message sequence charts and state transition diagrams. However, the support of these methods for verifying the internal aspects is weak because the generated prototype system visualizes external aspects only and does not have functions to achieve business logic. Therefore, the misunderstandings among the analysts for the internal aspects are hard to be cleared by each of the analysts. We try to improve this problem by visualizing the definition of the requirements specification about the internal aspects. Model checking techniques are useful for developers to validate a model based on specifications which are expressed as temporal logic efficiently and exhaustively. Such techniques are a promised approach to discover defects of the model early and sufficiently. Some researches try to detect defects of specifications or source codes (Aoki and Matsuura 2011; Choi and Watanabe 2005) by using model checking tools such as UPPAAL (UPPAAL 2010). (Choi et al. 2005) propose how to use the model checking technique for checking the consistency of a class specification and a page flow diagram, and for checking the consistency of the class specification and activity diagrams. The activity diagrams are a use-case-based behavioral model, so the activity diagrams contain brief definition for the internal aspects. However, this method can not verify the internal aspects because this method focuses on the checking for the external aspects. One of the essential difficulties for the model checking techniques is how the analysts define adequate temporal logic formulas. The consistency of the formulas is difficult to be kept by the analysts if the analysts define the formulas by distributed work with the misunderstanding of the way of design. To improve this problem, support of enhancing common understanding among the analysts is inevitable in order to share the understanding to the requirements specification precisely even if useful model checking techniques are used. Our approach aims for enhancing common understanding of the requirements specification for the analysts, and focuses on the internal aspects.

### Detailed specifications

The testing such as unit test or join test is normally performed in order to confirm the validity of the internal aspects. Such testing requires test cases, test data, and the source codes of a product of developers basically. Such low level artifacts often require detailed specifications so that the product can be executed. However, the cost of detailing specifications may come to nothing if such detailed specifications were created based on above-mentioned misunderstanding among the analysts. Therefore, we propose a way to review the UML RA model based on scenarios which imply test cases and test data for integration test without detailing the UML RA model. At least, the UML RA model should represent the data flow of essential data such as entities and the relation between actions and data, but the analysts do not need to detail the actions until the actions can be executed. Namely, the analysts concentrate their effort on defining the scenarios based on the UML RA model. There are reading techniques (Thelin et al. 2003) to discover defects on the documents such as requirements or design documents. (Thelin et al. 2003) proposed Use case Based Reading (UBR). The UBR is to find defects the documents created at upper process according to use cases prepared before using UBR. The objective of UBR is to efficiently discover defect of the documents which are ranked by selecting use cases. The UBR is more effective than Checklist Based Reading (CBR) to efficiently find defects within limited time. The use case should be correct to effectively use the UBR because the UBR relies on the use case completely. The focus is different between these methods and our proposal because our proposed review method focuses on the verification for the use cases not design documents. In the CBR, reviewers check the documents by the checklist which is created based on past experience of development. Therefore, the checklist tends to be generalized independent from each application so that reviewer can reuse to various application. However, such check-list sufficiently deals with defects on each application. Our proposed review method can deals with such defects by using object diagrams which represent concrete users’ expectation.

## The UML RA model

**Figure 1:**
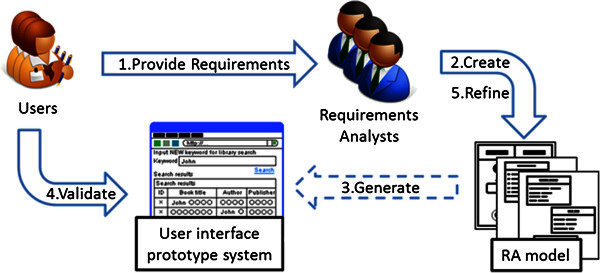
**The process of requirements analysis by using the UML RA model.** Analysts refine a UML RA model iteratively by making users validate the model. The users operate generated prototype system when they validate the UML RA model.

Figure 1 shows the process of requirements analysis by using the UML RA model. We have provided analysts with a CASE tool for generating the user interface prototype system so that the analysts can make the users validate the UML RA model intuitively and efficiently by using the generated prototype system. The CASE tool also enhances the efficiency of the iteration of users’ validation easily by automatically generating a user interface prototype system based on modified UML RA model in the iteration. Then, the analysts refine the UML RA model until the generated user interface prototype system meets the users’ expectation.

### UML diagrams in the UML RA msodel

**Figure 2:**
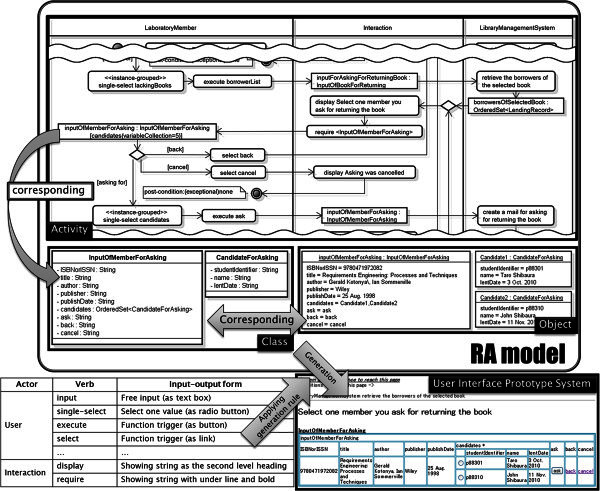
**An image of the generation of a user interface prototype system from the UML RA model.** Three kinds of diagrams are transformed into a user interface prototype system by using the rules of corresponding between UML elements and user interface components.

Figure 2 shows an example of an UML RA model and the generated user interface prototype system. This UML RA model depicts a use case “Ask For Returning A Book” of Library Management System (LMS). The top of the Figure 2 shows the UML RA model which consists of activity diagrams, a class diagram and object diagrams. In the activity diagram defined by analysts, there are three kinds of actors as partitions such as “User”, “Interaction” and “System.” In this example, the “User” implies “LaboratoryMember”, the “Interaction” implies “Interaction” and the “System” implies “LibraryManagementSystem.” The “User” and “Interaction” partitions represent the user-side process on user interfaces so that the users can understand the capability about users’ operation to the system. The series of actions of the “User” such as “single-select lackingBooks” represent operation steps for each user interface. The series of object nodes of the “User” such as “inputOfMemberForAsking” represent input/output data on each user interface.

The series of actions of the “Interaction” such as “require <inputOfMemberForAsking>” represent the system process for accepting or outputting the input/output data. The “System” partition represents the fundamental process of business logic so as to clarify the important aspects for interactive software such as the data flow of entities and the process for the entities. The series of actions of the “System” such as “retrieve the borrowers of the selected book” represent business logic briefly. The series of object nodes of the “System” such as “borrowersOfSelectedBook” represent entities handled by the business logic. In the left of Figure 2, the classes are assigned into the object nodes by the analysts so as to clarify the relation between a structure and objects. There are two types of objects in the activity diagrams. One is “entity” class whose objects appear at the partition of “System.” The “entity” objects are corresponding with the conceptual model represented as a class diagram. The other is “boundary” class whose objects appear at the partition of “User” or “Interaction.” The “boundary” objects are derived from the “entity” objects because the “boundary” objects exist for inputting or displaying the “entity” objects. In the right of Figure 2, the analysts define the object diagram for each object node so that the users and analysts can understand the value format and range of each structure enough.

### User interface prototype system

The right and bottom of Figure 2 shows a part of the user interface prototype system generated from above-mentioned UML RA model. In this generation, the elements of the “User” and “Interaction” partitions are transformed into elements of the generated prototype system. The actions are transformed into user interface components such as text boxes, radio buttons, buttons, links, etc. The left and bottom of Figure 2 shows the rules for transforming from the verb of an action to a user interface component. For example, the “single-select candidates” action is transformed into radio buttons named “candidates.” The class corresponding with object nodes is also transformed into a table expression so as to represent data structure. For example, the “InputOfMemberForAsking” class which is assigned to the “inputOfMemberForAsking” object node is transformed into the table named “inputOfMemberForAsking” in Figure 2. The instance specification corresponding with a class is transformed into a concrete instance for each attribute of the class. For example, the “Candidate1” instance specification is transformed to a concrete instance for the “candidates” attribute of the “InputOfMemberForAsking” class. In addition, a usage scenario can be also expressed by defining object diagrams for each object node on a path of the flow of activity diagrams. The analysts can represent the state change of the object in the path more exactly by the usage scenario so that the users can understand the UML RA model intuitively and easily. This scenario can be also transformed into the user interface prototype system.

## Method

**Figure 3:**
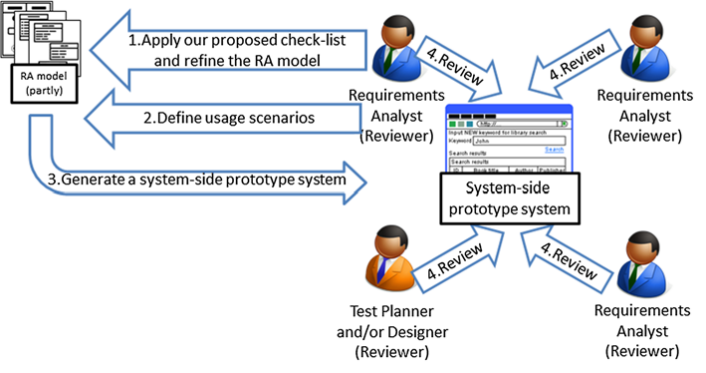
**The process of the review of the UML RA model by using our proposed method.** Each analyst firstly understands his/her part of the UML RA model about the internal aspects by using our proposed check-list. Then, all of the analysts review the entire model by using generated system-side prototype systems after each analyst refined his/her part of the model based on the check-list.

In this section, we propose a review method of the UML RA model about the internal aspects so that the analysts can verify the consistency among each diagram intuitively and efficiently. Each of analysts in a group analyzes a part of the UML RA model separated by a certain unit of behavior such as several use cases and functions with distributed work. Therefore, the review of the model is needed to verify the consistency among each model defined by different analysts because there are few cases that all of the analysts understand the entire model precisely. The internal aspects are also defined briefly to enhance its understandability for the users and analysts at an early stage of the software development (Cockburn 2000) but its definition is easy to become ambiguous. So, we assume two fundamental steps to review the model effectively with group work. The first step is that each of analysts understand his/her own model in distributed work because he/she can not recognize the inconsistency between other parts of the model and his/her own model if he/she does not understand his/her own model. Here, the inconsistency implies mismatches of pre/post-conditions between actions, and inadequate relations between an action and objects. Then, the second step is that all of the analysts understand each part of the model so that each analyst can recognize the inconsistency among each part of the model easily and intuitively. By the way, the usage scenario is a concrete example which shows the result of inserting concrete data into each object node so as to fit the pre and post conditions of each action. Therefore, we assume that the analysts can recognize the inconsistency sufficiently by using the usage scenarios. Our proposal consists of two artifacts for supporting analysts’ verification of the internal aspects defined in the UML RA model. The two artifacts are also considered so as to support above-mentioned two steps of the review. One is a check-list for understanding the definition of internal aspects by each analyst so that each analyst can understand own definition precisely by self. The other is a system-side prototype system for visualizing the result of integrating three kinds of the diagrams in the UML RA model so that the analyst can understand the entire UML RA model efficiently and intuitively. Here, the three kinds of the diagrams are activity diagrams, a class diagram and object diagrams, which imply the usage scenarios. Figure 3 shows the process of the review of the UML RA model by using our proposed method. Figure 3 implies the process after performing the process of Figure 1. We describe the steps of the review as follows.

1. Each analyst applies our proposed check-list to each part of the UML RA model, and refines his/her part of the UML RA model based on the check-list. Here, it has few problems if the UML RA model as the premise was defined by other analysts who do not use our proposed review method. But, if there are unclear definitions, the analysts who review the model may interact with the original modeler to clarify those definitions. The analysts should also target the UML RA model after finishing the refinement of the model based on users’ validation because the structure of the operation steps strongly influences the internal aspects.
2. Each analyst creates usage scenarios in order to check whether the UML RA model contains defects in the range of his/her part. He/she firstly clarifies unclear actions, then checks whether the inconsistencies are contained.
3. After each analyst corrects such defects of his/her part, each analyst generates each system-side prototype system from his/her part of the UML RA model by using our proposed CASE tool.
4. All of the analysts as the reviewers verify the entire UML RA model. In addition, designers and/or test planners may participate into the review in order to support confirming the feasibility of the model.

As the result of applying our proposed method to the review of a UML RA model, it is expected that the designers and test planners can obtain the model which does not contain serious inconsistency and which enhances the assurance of the feasibility of the model. And such model can be obtained efficiently because the analysts do not need to detail UML RA model. The analysts need to define the usage scenarios but we think that this cost is inevitable because this definition has the same meaning of the test planning.

### Check-list for precise understanding of the UML RA model

**Figure 4:**
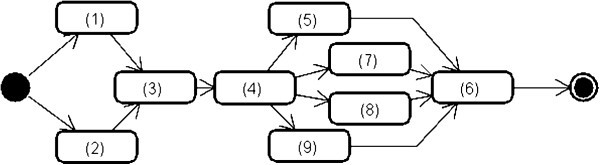
**The order of applying the check points to the UML RA model.** This shows the order to applying the check-list in Table 1 to the checking of the internal aspects of the UML RA model.

**Figure 5:**
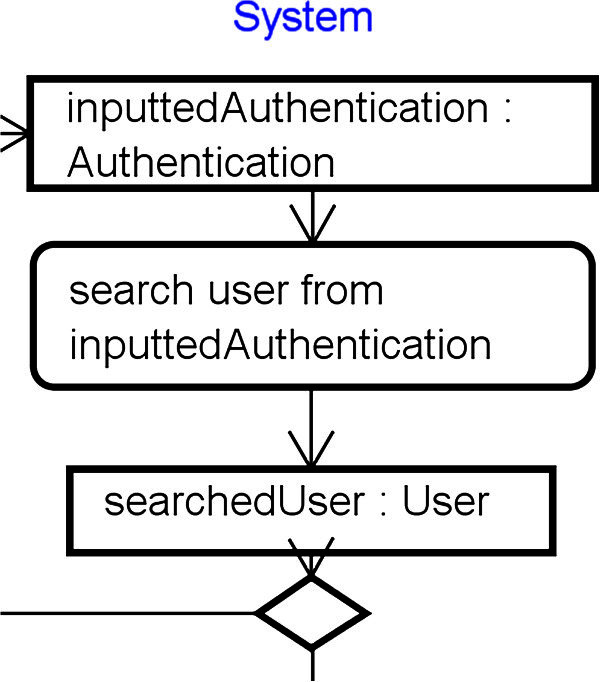
**A part of a function of login.** This is a simple example and shows the internal part of the function of login.

Generally, analysts divide huge tasks into small set of the tasks, then each of the analysts do each set of the tasks as group work in a large-scale software development. The use case is often used as a unit of distributed work at an early stage of a software development. Our proposed check-list is applied to such situation with distributed work. The check-list is used for understanding the internal aspects in the UML RA model. As the result, it is expected that unclear actions and the inconsistencies such as mismatch of pre/post conditions between actions, and inadequate relations between an action and objects are discovered. Table 1 shows all of the check items we propose. This check-list lets analysts express the properties of each element by considering the usage scenarios. In Table 1, “Diagrams” implies the kinds of UML diagram in the UML RA model. “Check Points” are the queries to be answered by each analyst for his/her part of the model. “Elements” implies that the analysts should focus on the basic elements to check based on the “Check Points.” “Properties” implies that the analysts should focus on the properties of the focused basic elements. Figure 4 also shows the order of applying the check points to the UML RA model. (1) of the “Check Points” implies that the action which needs input objects represents the name of the input objects in the contents of the action. (2) also implies that the action which need needs an output object represents the object node immediately after the action. An example of satisfying these two points is shown in Figure 5. Figure 5 depicts a part of a function of login. The action “search user from inputtedAuthentication” needs “inputtedAuthentication” as the input object. And “inputtedAuthentication” is corresponding with the object node “inputtedAuthentication: Authentication.” Therefore, this action satisfies (1). This action also contains the verb of “search.” Generally, the “create” and “read” actions outputs created/read object. And the object node “searchedUser : User” is located immediately after the action. Therefore, this actions satisfies (2).

**Table 1:** **Our proposed check-list for precisely understanding the UML RA model**

Diagrams	Check points	Elements	Properties
Activity diagram	(1) Are input objects of actions expressed?	Action	Name
	(2) Are output objects of actions expressed?	Action, Object node	Location
	(3) Are derived relations between objects expressed?	Object node	Derived relation
	(4) Are pre/post conditions of actions expressed?	Action	Pre/post condition
	(5) Are there inappropriate objects about singular/collection?	Object node	Classifier, Name
	(6) Are conditional branches omitted?	Decision node	Guard
Class diagram	(7) Is each range of values expressed?	Attribute of class	Invariant
	(8) Is each format of values expressed?	Attribute of class	Invariant
	(9) Are there inconsistencies between a type and values?	Class, Instance spec.	Type of attribute, Value of slot

This check-list is used by each analyst to understand his/her part of the UML RA model about the internal aspects precisely.

**Figure 6:**
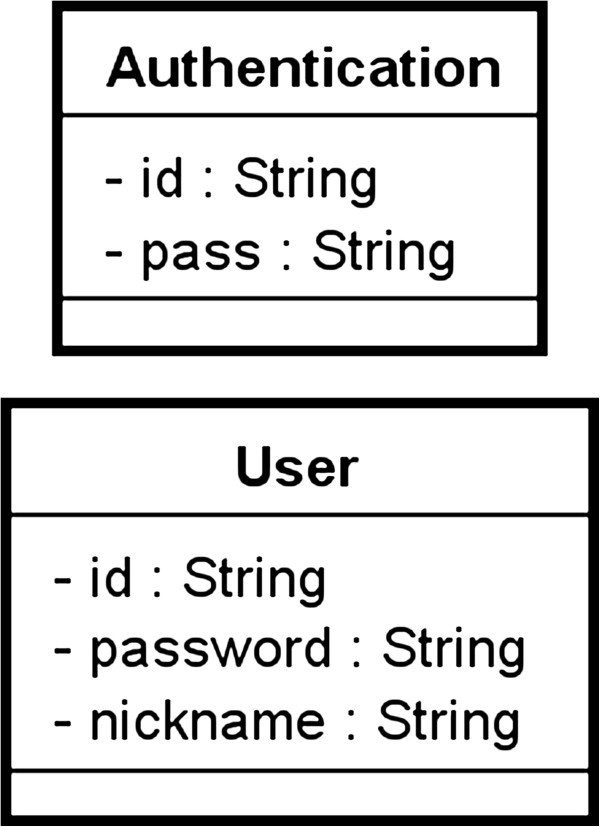
**A part of the class diagram related to the function of login.** These classes are entities related to the function of login.

Figure 6 shows a part of the class diagram related to above-mentioned function of login. (3) implies that the derived relations between related object nodes each other are decided based on the attributes of the classes. The object nodes of “inputtedAuthentication” and “searchedUser” have the following relations: inputtedAuthentication.id == searchedUser.id; inputtedAuthentication.pass == searchedUser.password. It is important for the analysts to understand these relations precisely rather than write these relations rigorously. These relations will be finally defined as formal language such as Object Constraint Language (Object Constraint Language 2012) after the relations were fixed by using our proposed review method. (4) implies that pre/post conditions of each action are decided based on above-mentioned derived relations. For example, the pre-condition of the action “search user from inputtedAuthentication” is to need the object “inputtedAuthentication.” And the post conditions of the action is to read the object “searched User” so as to satisfy the derived relations mentioned at (3) in all instances of “User” class of the system.

Then, (5), (7), (8) and (9) are considered by the analysts based on the results from (1) to (4). (5) implies checking the validity of the type of classes. In the case that a class is the collection type but the analysts misunderstand the class as the singular type, it takes expensive cost for modification afterwards. (7) and (8) imply checking the invariants of attributes of classes. These invariants can be decided by seeing common properties from above-mentioned derived relations and the concrete values of usage scenarios. (9) implies is verifying the consistency between a type and values. If concrete value is “5 Apr. 2012” and its type is Integer, the type or value is inadequate because the value is the type of String. Finally, (6) implies discovering omissions of conditional branches. These omissions are discovered by seeing the pre/post conditions of actions basically. Although there are pre/post conditions of actions, that is the omission of guards of the flow if the model does not consider the case of that exceptional flows lack for some of the pre/post conditions.

### System-side prototype system

**Figure 7:**
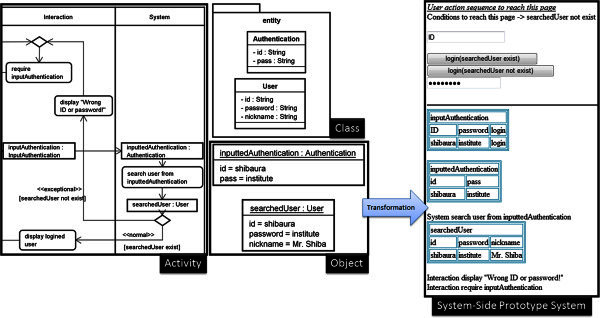
**An example of the system-side prototype system generated from the internal aspects defined in the UML RA model.** This prototype system is not functional one but offers support of enhancing the ease of understanding to the analysts because three kinds of diagrams are integrated to a kind of Web pages.

Figure 7 shows an example of the system-side prototype system generated from the internal aspects defined in the UML RA model. This prototype system aims for visualizing the invisible aspects for the users. The analysts can understand the internal aspects intuitively and efficiently because the system-side prototype system shows the result of integrating three different types of diagrams such as activity diagrams, a class diagram and object diagrams shown in Figure 7. The way to transform the UML RA model into the system-side prototype system is constructed based on the algorithm of the transformation of the user interface prototype system basically. In the system-side prototype system, user interface components are shown above the horizontal line. The actions of the system are transformed into text messages such “System search user from inputtedAuthentication” simply.

## Results and discussion

We have evaluated the effectiveness of our proposed review method through a case study. As the result, serious inconsistencies were discovered by using our proposed review method through a software development of Library Management System (LMS) for a small laboratory.

### Participants and process

There are six participants for this case study. One participant plays a role of the user of LMS, and another participant plays a role of the primary analyst to create initial RA model. The rest of participants play roles of the reviewers and secondary analysts. An initial UML RA model was created by the primary analyst. He performed the process in Figure 1. Then, the secondary analysts refine the initial UML RA model using our proposed review method. In this evaluation, the primary analyst understood the entire UML RA model but each of the secondary analysts understood his/her part of the UML RA model until all of the secondary analysts review the entire UML RA model by using the generated system-side prototype systems for each part of the UML RA model. The initial UML RA model was also manually verified by the primary analyst because he wants not to add the defects into the RA model. The primary analyst did not use any systematical methods for the review of the UML RA model. Therefore, the effectiveness of our proposed review method is shown if the secondary analysts can discover defects of the initial UML RA model by using our proposed review method, because the primary analyst could not discover defects sufficiently by the heuristic way of him to review. There are also three characteristics of this case study to emphasize the versatility of our proposed review method. One is that the reviewers are not the creator of the initial UML RA model i.e. the reviewers do not completely understand the entire UML RA model at first. Second is that three of four secondary analysts are the novices to use the UML RA model because the three secondary analysts have not ever dealt with the UML RA model. Third is that the three secondary analysts had few experience of requirements analysis because they are bachelor students. The scale of the UML RA model of the LMS is shown in Table 2.

**Table 2:** **The scale of the UML RA model of LMS**

Elements	Number
Use case	5
Class(Boundary)	27
Class(Entity)	6
Action	207
Activity flow	406
Usage scenario	114
Object diagram for all of the usage scenarios	1154
Instance specification for all of the object diagrams for all of the usage scenarios	4331

This shows the scale of the UML RA model from the viewpoints of the number of elements.

### Results and consideration

**Figure 8:**
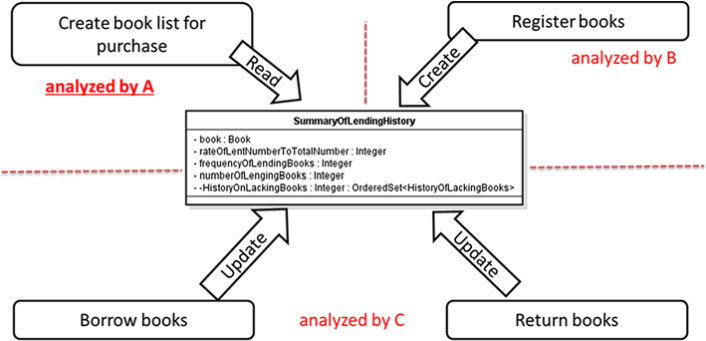
**The responsibility of use cases assumed by A.** This shows the assumption of A for the UML RA model of the LMS.

**Figure 9:**
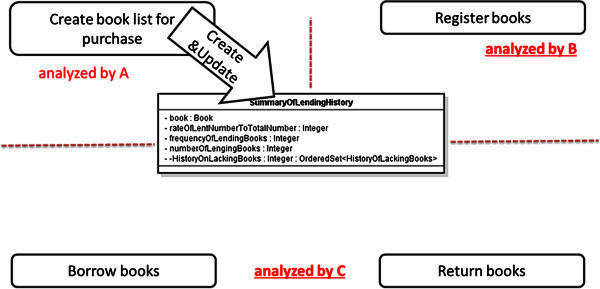
**The responsibility of use cases assumed by B and C.** This shows the assumption of B and C for the UML RA model of the LMS.

**Figure 10:**
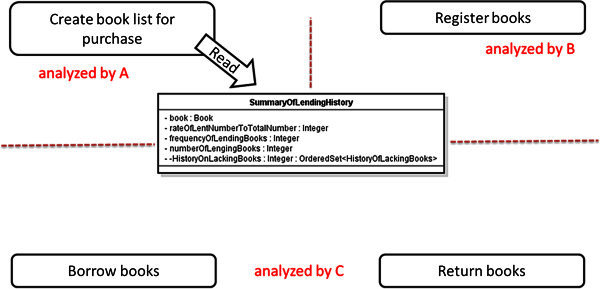
**Actual definition of the UML RA model.** This shows an actual definition of the UML RA model of the LMS.

As the result of this case study, the number of the defects which the secondary analysts discovered by using our proposed check-list was 44 as total. The time cost is 39 man-hours for defining all of the usage scenarios. On the other hand, the time cost is also 36 man-hours for the review. The secondary analysts also discovered the serious inconsistency caused by the misunderstanding among the analysts for the use cases, by using our proposed system-side prototype system. Figure 8 shows assumption of the responsibility of use cases to a class “SummaryOfLendingHistory” by A. Figure 9 shows the assumption by B and C. Figure 10 shows the UML RA model defined actually. Here, A, B and C mean three of the secondary analysts.

The class “SummaryOfLendingHistory” represents a history of borrowing or returning of a book. Therefore, it seems that the life-cycle of this history is corresponding to the life-cycle of the book which is used by several different use cases such as “Create book list for purchase,” “Register books,” “Borrow books” and “Return books,” as shown in Figure 8. On the other hand, these histories are used in only one use case “Create book list for purchase” so that the users can discover the lacks of books, then choose the books for purchase. Therefore, it also seems that the life-cycle of this history is closed completely in the use case “Create book list for purchase” because these histories are used by only this use case, as shown in Figure 9.

On this background, the serious inconsistency was caused as shown in Figure 10 by the misunderstanding between the second analysts. Figure 10 implies that the instances of “SummaryOfLendingHistory” can be read although any instances of the class can not be created. Such serious inconsistencies jeopardize the feasibility of the UML RA model. Our proposed review method contributed for discovering such inconsistencies. The enhancement of the efficiency of the review by using our proposed method is explained without detailing the model. And the effectiveness of our proposed method is explained by that the inconsistencies were discovered by novice analysts through the system-side prototype system.

## Conclusion

We proposed a review method to efficiently discover serious inconsistencies manually at an early stage of a software development focusing on the brief definition of the internal aspects. As the result of the case study, our proposed review method contributed to discover serious inconsistencies which make the developers impossible to implement the product based on the model. If such inconsistencies are overlooked, disastrous reworking will be caused at implementation or testing phase. As future work, we consider to automate a part of check items in our proposed check-list. For example, the validity of relations between an action and an object node can be systematically decided by using the notation of UML. We also consider a way to detail the conditions such as pre/post-conditions gradually based on our proposed check-list.
